# Pyeloureteral Junction Syndrome in a Neonate With a Solitary Kidney Treated by Anderson-Hynes Pyeloplasty: A Case Report

**DOI:** 10.7759/cureus.65589

**Published:** 2024-07-28

**Authors:** Ismail Benomar, Souha Qarouach, Mohamed Rami, Rachid Belkacem, Mohamed Amine Bouhafs

**Affiliations:** 1 Pediatric Surgery, Children's Hospital Faculty of Medicine and Pharmacy, University Mohamed V, Rabat, MAR

**Keywords:** pyeloplasty, anderson hynes, pyeloureteral junction, hydronephrosis, new born

## Abstract

A rare disorder called newborn hydronephrosis is mostly caused by the obstruction of the pyeloureteral junction. We describe a case study of a male neonate who underwent Anderson-Hynes pyeloplasty to effectively cure hydronephrosis in a single kidney that was further complicated by early renal failure.

Considering kidney failure can result in progressive renal fibrosis, early management is essential. The gold standard is Anderson-Hynes pyeloplasty, which is usually advised for individuals who weigh over 10 kg. Percutaneous nephrostomy is frequently used in the early stages of care for newborns in order to reduce dilatation and restore renal elasticity. After surgery, the resolution of hydronephrosis may take up to 24 months. We note that pyeloureteral junction obstruction can appear as a single anomaly or a component of a multifactorial illness.

This study aims to contribute to the discourse surrounding the optimal timing of Anderson-Hynes pyeloplasty in pediatric patients, providing insights into clinical management strategies and outcomes.

## Introduction

Specialists frequently refer to a distension of the pelvis and calyces caused by a blockage as "hydronephrosis," which, if left untreated, causes the kidneys' condition to gradually worsen. How to classify dilatation of the prenatal and postnatal urinary systems is still up for debate. Pyeloureteral junction blockage is the most common cause of hydronephrosis, accounting for 1 in 1000-1500 cases, according to estimates [1].

Most often, pyeloureteral junction obstruction is understood to be a functional obstruction resulting from anomalies in the ureteric and pelvic smooth muscles. The pyeloureteral junction blockage is also observed when crossing vessels, however, it is still up for debate whether the obstruction is caused by the vessel alone or if there is a functional component as well [1].

This work aims to contribute to the discussion on the ideal age for pyeloplasty using the Anderson-Hynes technique.

## Case presentation

**Figure 1:**
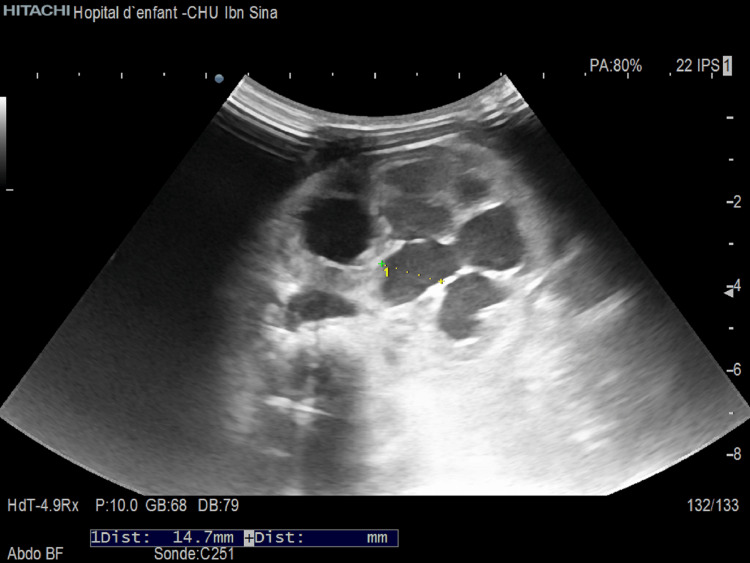
Anteroposterior diameter of the pyelon measured at 14.7 mm

**Figure 2:**
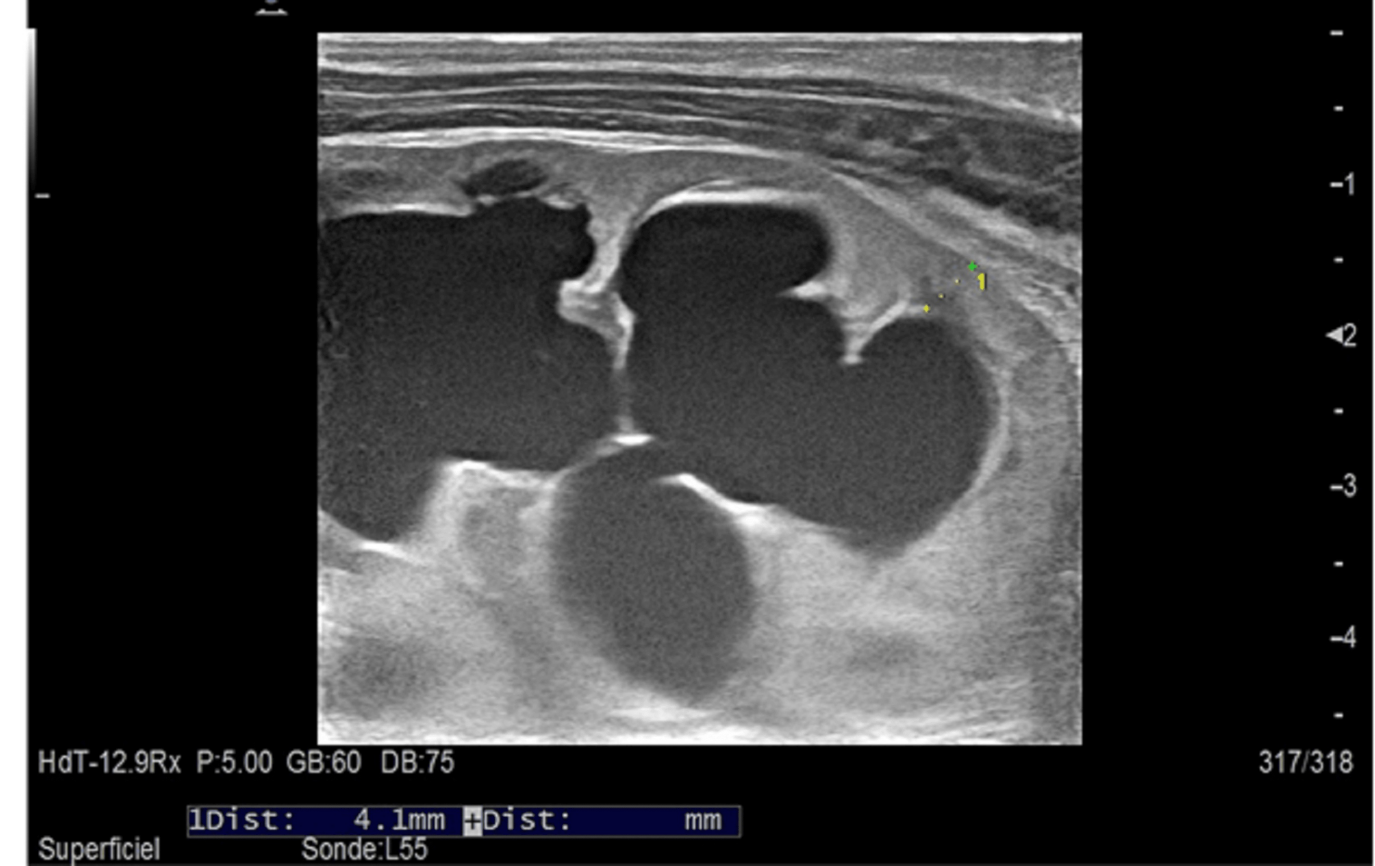
Significant dilatation of the calyceal cavities laminating the renal parenchyma measured at 4.1 mm, with persistent cortico-medullary differentiation

The newborn patient was a 15-day-old male, born to a 39-year-old primigravida mother, who had undergone an antenatal ultrasound with evidence of minimal hydronephrosis of the right kidney. He presented with vomiting of milk and a refusal to feed for two days. The newborn wet his diapers during the first days of life as reported by the family, with a decrease in the frequency of diaper changes until total anuria two days before admission. On admission, the newborn was slightly hypotonic, with no signs of dehydration or malnutrition, and no break in the weight curve (weight=3100g), blood pressure = Systol: 78 mmHg, Diastol: 35 mmHg, no bladder globe, no mass, and no other malformations on clinical examination. Abdominal ultrasonography was performed as the first-line exam and was suggestive of a pyeloureteral junction syndrome in a single right kidney with an anteroposterior diameter of the pyelon measured at 14.7 mm (Figure 1), as well as significant dilatation of the calyceal cavities laminating the renal parenchyma measured at 4.1 mm, with persistent cortico-medullary differentiation (Figure 2). The kidney was hypertrophic (Length=56 mm, Width=40 mm, Thickness=35mm). The biological profile was as follows: kalemia > 7 mEq/l, natremia 117 mEq/l, alkaline reserve 11 mEq/l, urea 0.20 g/l, creatinine 27.1 mg/l (creatinine clearance 8.37 ml/min) (Table 1).

**Table 1:** Biological profile of the patient

Parameters	Results	Reference range
Kaliemia	>7 mEq/l	3.5 - 5.10 mEq/l
Natremia	117 mEq/l	136 – 145 mEq/l
Urea	0.20 g/l	0,10 – 0.36 g/l
Creatinine	27.1 mg/l	5.7 – 12.5 mg/l

**Figure 3:**
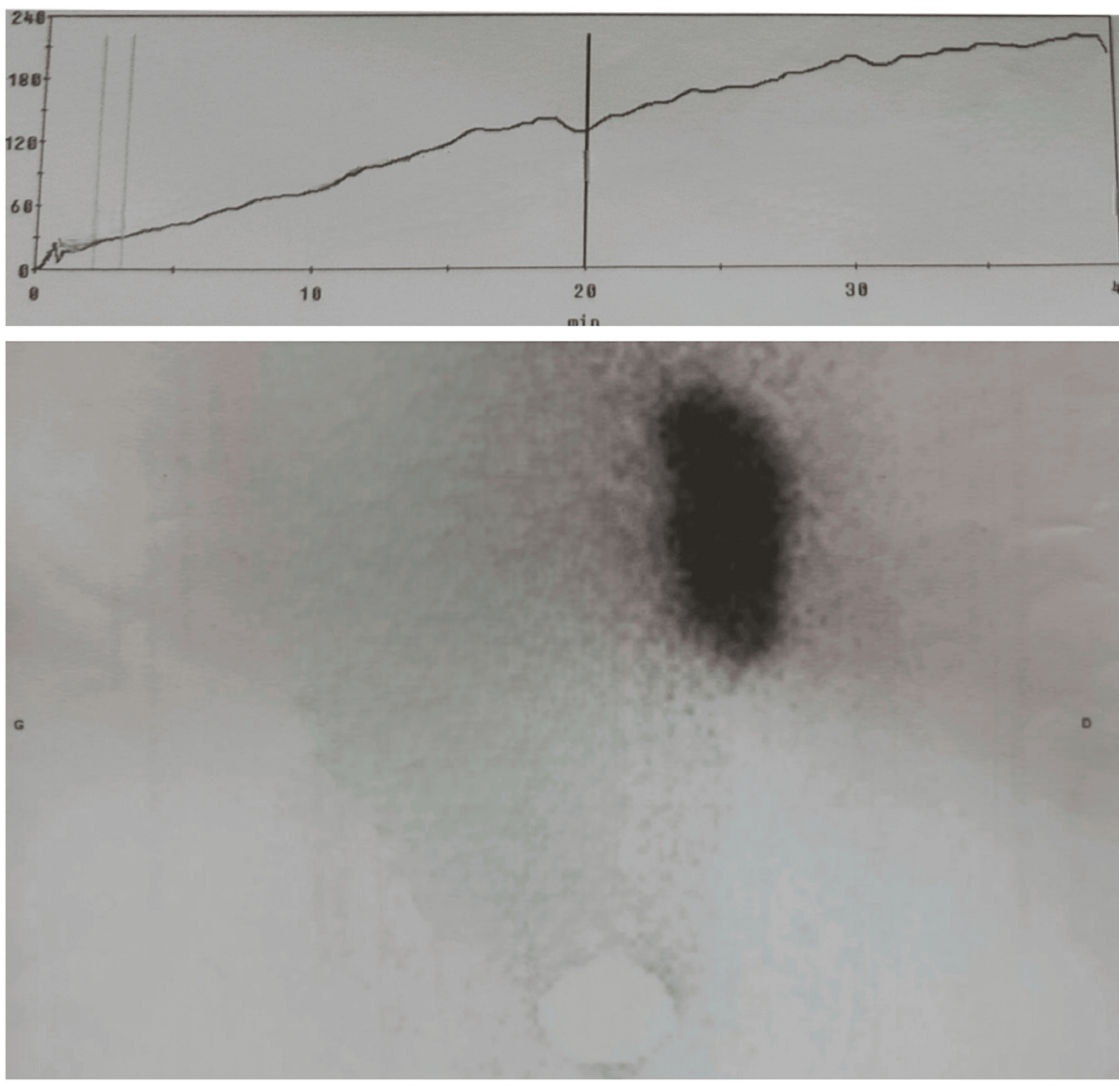
MAG3 renal scintigraphy revealing impaired renal excretion MAG3: mercaptoacetyltriglycine

MAG 3 (mercaptoacetyltriglycine) renal scintigraphy was performed, showing 100% renal function on a single kidney and poor drainage (Figure 3).

The patient was discussed with the nephrology and anesthesiology teams, and the decision to proceed with surgery was made after correction of the hydroelectolytic imbalance.

The patient was admitted to the operating room under general anesthesia, intubated and ventilated, and placed in the left lateral decubitus position. An incision was made midway between the twelfth rib and the iliac crest, and after approaching the retroperitoneal region, we found a dilated pylon and a thin ureter, suggestive of a constriction of the pyeloureteral junction on a congenital bridle.

The surgical procedure involved resecting the area of caliber discrepancy and performing a pyeloureteral anastomosis using the Anderson-Hynes technique. Subsequently, a transurethral catheter, Redon drain, and Foley urinary catheter were inserted.

The postoperative course was straightforward, with improvement in renal function. An ultrasound at eight days postop showed a reduction in calyceal dilatation. Electrolyte balance was normalized at seven days postop, with kalemia 4.8 mEq/l, natremia 134 mEq/l, urea 0.10 g/l, and creatinine 4.3 g/l (Table 2). No proteinuria was detected.

**Table 2:** Postop biological profile

Parameter	Results	Reference Range
Kaliemia	4.8 mEq/l	3.5 - 5.10 mEq/l
Natremia	134 mEq/l	136 – 145 mEq/l
Uréa	0.10 g/l	0,10 – 0.36 g/l
Creatinine	4.3 mg/l	5.7 – 12.5 mg/l

The trans-anastomotic catheter was removed on Day 15 postop while the Foley urinary catheter was removed on Day 20 after ensuring correct diuresis and the absence of stenosis or leakage of the anastomosis (note that the probe was changed regularly to avoid the risk of infection).

The patient was followed up weekly for one month with clinical and biological follow-up, then monthly. The 11-month follow-up showed that renal function and residual pyelon dilatation had stabilized.

## Discussion

Hydronephrosis of the newborn is a rare pathology often linked to stenosis of the pyeloureteral junction, with one new case in 1000-1500 births [2,3]. The impact of hydronephrosis begins in the fetal period and involves ischemic and apoptotic mechanisms, tubular and glomerular atrophy, and progressive fibrosis [4].

Pyeloplasty using the Anderson-Hynes technique is the gold standard for the treatment of pyeloureteral junction syndrome, with a 90% success rate [5]. Indications for pyeloplasty are a symptomatic pyeloureteral junction restriction (recurrent pain and infection), altered renal function on MAG3 scintigraphy with a >10% decrease in function on armed follow-up, or renal function <40% on the affected kidney [6]. Open or laparoscopic pyeloplasty may be considered from a weight of 10 kg [6]. During the neonatal period, percutaneous nephrotomy is favored to ensure that the patient is properly prepared for pyeloplasty, allowing a sufficient degree of restoration of the elasticity of the dilated tissues [7]. In our case, we opted for pyeloplasty from the first line, as the patient had a single kidney, impaired renal function, and relatively moderate pyelon dilatation.

A study including 125 patients who had undergone pyeloplasty showed that resolution of hydronephrosis can be achieved up to 24 months after surgery. While no single factor has been established to explain the delay in the resolution of hydronephrosis, several studies have suggested that risk factors may explain the delay [6]. Moreover, there is no consensus on the definition of the resolution of hydronephrosis. Carpenter et al. consider an anteroposterior diameter = 0 mm [8], Rickard et al. define resolution as an anteroposterior diameter <15 mm [9], while Sanni Varela et al. have opted for a diameter <10 mm or a decrease of more than 50% compared with the preoperative dimension [6].

It should be noted that there are syndromic forms of the pyeloureteral junction syndrome, which are part of several syndromes including g VACTERL, Schinzel-Gidion syndrome, Johanson-Blizzard syndrome, and Ochoa syndrome or trisomy 13/18 [7].

## Conclusions

Neonatal hydronephrosis is a rare entity caused essentially by pyeloureteral junction syndrome. In the neonatal period, it is preferable to opt for minimalist treatment with nephrostomy followed by pyeloplasty using the gold-standard Anderson-Hynes technique. Resolution of hydronephrosis can be achieved after an average of 24 months. We report a case of pyeloureteral junction syndrome in a newborn on a single kidney with pyeloplasty from the outset, with good clinical and biological evolution.
